# Empowering Maasai women behind the camera: Photovoice as a tool for trachoma control

**DOI:** 10.1186/s40900-021-00286-x

**Published:** 2021-07-05

**Authors:** Tara B. Mtuy, Jeremiah Mepukori, Joseph Lankoi, Shelley Lees

**Affiliations:** 1 Department of Global Health and Development, London School of Hygiene & Tropical Medicine, 15-17 Tavistock Place, London, WC1H 9SH UK; 2 ECLAT Development Foundation, Arusha, Tanzania; 3 Independent Researcher, Longido, Tanzania

**Keywords:** PPI, Photovoice, Trachoma, Maasai, Empowerment

## Abstract

**Background:**

Photovoice is a method used to help engage community members to understand local realities and promote social change. Photovoice uses cameras in the hands of participants as a tool to visually document a specified topic. Photos taken by participants allow for reflection and stimulate dialog on an issue to ideally lead to social change. Trachoma, hyperendemic in Maasai communities in Northern Tanzania, is the commonest infectious cause of blindness worldwide, caused by *chlamydia trachomatis.* The bacterial infection commonly occurs in childhood and over many years repeated infections leads to inflammation and scarring of the eyelid. Often as adults this leads to the upper eyelid turning inward and eyelashes scratching the eye, resulting in pain and eventually blindness. We used photovoice as a tool for Maasai women to share their lived experiences of educating peers on trachoma and ultimately empowering women in this society.

**Methods:**

This public engagement intervention was conducted September thru October 2017. We held a workshop on trachoma control for 20 Maasai women including use of photovoice method. Women were asked to disseminate information from the trachoma control workshop to their community and to capture their experiences using disposable cameras. Five weeks post-workshop we facilitated a discussion and women displayed photos of the successes and challenges they encountered as advocates for trachoma control in their community.

**Intervention Outcomes:**

It was observed throughout the process and at the photo discussion meeting, that women articulated empowerment by this experience; as educators, agents of change and a source of valued information.

**Conclusion:**

Photovoice should be considered for future interventions as a communication tool on health issues and to empower women to be ambassadors for health promotion.

## Plain English summary

Trachoma is a blinding eye disease caused by bacterial infections. It mostly effects people living in low- and middle-income countries including the Maasai tribe in Tanzania. Photovoice is a method using photography to document, reflect upon and engage community members to communicate issues of concern. This paper describes the use of photovoice as part of a program to educate Maasai women on preventing and managing trachoma in their community. In this intervention, co-developed with the community, participants were asked to share information from a workshop on trachoma with their community and to capture their experiences as educators using disposable cameras. Five weeks post-workshop women shared photos of the successes and challenges they encountered when educating the community about trachoma. Women demonstrated empowerment as participants in this public engagement intervention. The lessons learned from this intervention and its use of photovoice can inform future interventions for health promotion and to empower women to be ambasadors of change in resource limited settings.

## Introduction

Photovoice “is the process by which people identify, represent and enhance their community through a specific photographic technique” [1]. It enables community members to record and share their realities about a phenomenon through their own photography. Visual images are used to promote a participatory means of sharing knowledge, perceptions and experiences. They act as a tool to enable people to more critically think about community and open up discussion around social and political influences in their lives [2].

Photovoice puts cameras in the hands of participants as a tool to visually document a specified issue. Participants take photos or videos to capture the issue in their everyday lives to document how they interpret the issue. Photos taken by participants are reviewed either individually or in a group to allow for reflection and stimulate dialog to ideally lead to social change. Photovoice is used in research, development work and interventions. It is a method increasingly being used in research of marginalized populations to explore and address health inequities [3–6] and inform policy. Photovoice is a means of generating knowledge as a needs assessment tool to provide researchers or development programs with “the possibility of perceiving the world from the viewpoint of the people who lead lives that are different from those traditionally in control of the means for imaging the world” [7]. This brings forth the concerns from the community themselves rather than what the researcher or programs think is important. As an intervention, photovoice is an alternative participatory method to share and disseminate knowledge. Other methods of obtaining and sharing information include interviews or focus group discussions. These methods have the possible limitation of power differentials between the researcher and participants and Maasai may not well articulate their experiences and perspectives through these methods, possibly explained by a history of not feeling authorized to offer critiques to ‘outsiders’. Photovoice helps break down the power differential between researchers and the researched [5] as well as programs and the community by giving them more autonomy in the data collection process.

This paper describes a public engagement intervention of which one component was photovoice, a participatory method used with Maasai women to share their lived experiences of educating peers on trachoma and ultimately empowering women in this society.

### Context

Trachoma is the commonest infectious cause of blindness worldwide, caused by the bacteria, *chlamydia trachomatis.* The clinical features of trachoma are divided into those related to ‘active’ disease which is characterized by repeated infections and are most common in children under 10 years; and those associated with scarring. Early stages of trachoma are characterized by follicles and inflammation in the conjunctiva of the upper eyelid. Over time, contraction of scar tissues causes eyelids to turn inward. Eventually eyelashes may touch the eyeball, trichiasis, leading to blindness [8].

Trachoma is a major public health concern in Maasai communities in Northern Tanzania. In 2016 the prevalence of trachoma in predominantly Maasai districts in Northern Tanzania was more than 50% [9]. Risk factors for trachoma include limited access and use of water [10]; limited face washing [11–13]; poor sanitation [14, 15]; and crowding [16]. Residents of Maasai communities in Tanzania and Kenya have shown a limited biological and public health knowledge of trachoma, transmission, prevention and control measures. Despite experiences and awareness of trachoma as well as an indigenous understanding, there was poor understating of aetiology and prevention [17, 18]. Control of trachoma is based on the World Health Organizations (WHO) SAFE Strategy composed of four public health interventions: Surgery for trachomatous trichiasis; Antibiotic treatment to eliminate the infection; Facial cleanliness promoting hygiene to reduce transmission; and Environmental change which includes management of human and animal feces, cleanliness to reduce flies, crowding and access to water [19]. Despite the Tanzania National Neglected Tropical Disease (NTD) Control Programme coordinating the delivery of improved SAFE strategies in these communities, trachoma remains a concern in Maasai districts in Tanzania. Mass drug administration (MDA) of antibiotics has challenges including logistics in the environmental terrain of pastoralist societies but additionally socio-cultural factors and political history influencing perceptions of the programme. A study looking at MDA in a Maasai community in Tanzania found that norms around pregnancy led women to accept the antibiotic but hide their refusal to swallow the drug. The timing of drug distributor visits conflicted with livestock grazing and women attending to household chores such as fetching water or firewood. Refusals occurred among the ilmurrani age group (young adult men) due to cultural norms related to creating strong bonds within their age group. Mistrust significantly hindered uptake of drugs possibly due to a history of political and social subjugation [20]. Land tenure policies in Maasai communities created during colonialization, many of which still exist today, were an effort to consolidate and isolate the Maasai and their cattle into designated areas and restricting their movement and interactions outside those areas [21]. Maasai questioned the government’s efforts to distribute antibiotics for trachoma against what they saw as important yet neglected priorities for resources for hospitals, medicines, clean water and roads [20].

The lead researcher (TBM) conducted ethnographic field work in Northern Tanzania from September 2016 to December 2017 [17, 20]. The purpose of the research was to document the community’s understanding of trachoma and responses to the National NTD Control Programme for trachoma elimination. In an effort to disseminate accurate information on trachoma following the research, a public engagement intervention using photovoice method was conducted in the same research community by the lead researcher. The National Co-ordinating Centre for Public Engagement in United Kingdom defines public engagement as the myriad of ways in which activity and benefits of higher education and research can be shared with the public. Engagement is a two-way process, with a goal of generating mutual benefit [22]. This paper describes the experience of women participating in the public engagement intervention, ways in which women were enabled to learn and to be teachers around trachoma within their community and ultimately empowering women in this society.

While this project could be used to inform health education, the primary purpose of photovoice in this project was as an intervention to visually document the outcomes of a health education program. Measuring the translation of knowledge into practice has challenges and takes time. Use of photovoice to visually document women’s efforts to transfer knowledge into practice aimed to (1) provide an alternative method to other qualitative methods to reflect and share experiences and challenges of trachoma control methods; (2) shift power to the women by allowing them to be instrumental in the data collection process and motivate them to fully participate; and (3) measure success of the intervention. This project put Maasai women behind the camera, to use photography as a means to describe their role as advocates for trachoma control and its impact.

## Methods

**Fig. 1:**
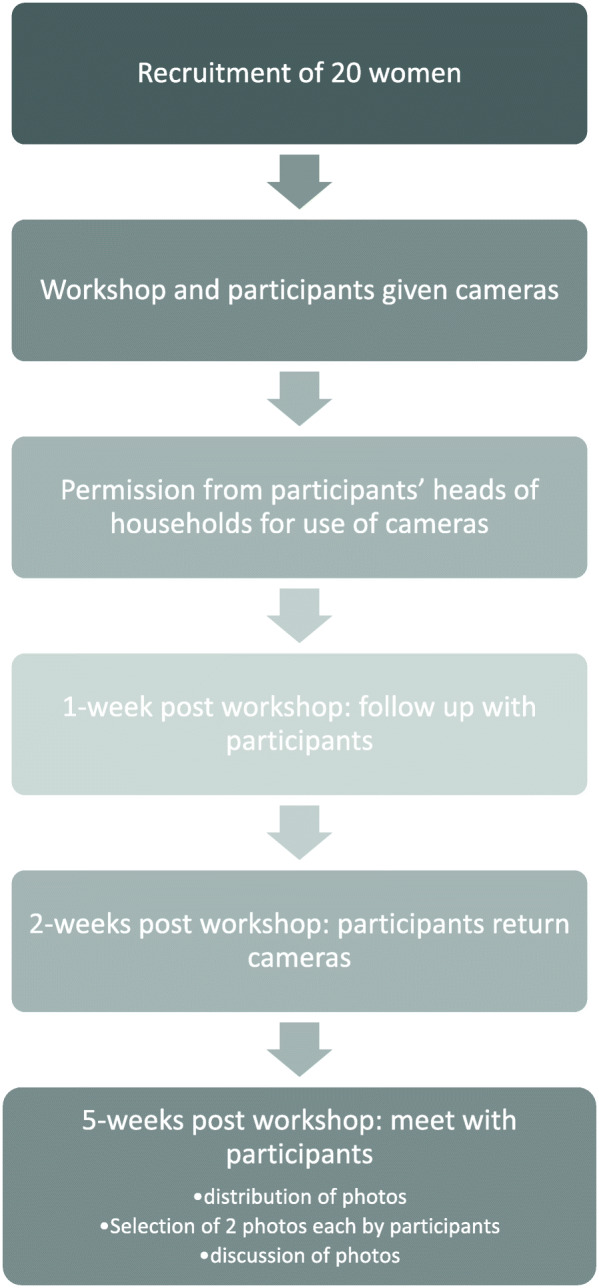
Flow of photovoice activities

This was a public engagement intervention for Maasai women, aimed to raise awareness about trachoma infection and control measures in a trachoma endemic community in Tanzania. The intervention consisted of a workshop which included photovoice training, participants disseminating knowledge from the workshop within their sub-villages, documentation of trachoma control in the community through photography and ending with a group discussion of the participants’ photos. The flow of the project is shown in Fig. 1. Findings were based on observations during the intervention, follow up and discussions during the photo sharing workshop.

The aim was to co-design the intervention and give power to the community to be actively involved in aspects of the intervention. Local leaders and lay Maasai men were consulted for various aspects of the intervention design: recruitment of women, consent, plans for follow up post-workshop, logistics of collecting cameras, and distribution of photos to women. Men are primarily involved in official planning due the gendered hierarchical system in Maasai culture. Yet the research team informally included women’s suggestions in the intervention. Women requested during the workshop, that the researchers introduce the photography aspect to their husbands and heads of household which was implemented.

The participants were 20 Maasai women coming from three villages, Il Donyo, Leremeta, and Endonyoemali, in Northern Tanzania. The three village chairmen selected two women in each sub-village (total of 10 sub-villages) to participate in the intervention and contacted them directly. In Maasai communities the village chairmen should be included in any decision-making regarding programs in their village. Typically, communities hold a lot of respect for village chairmen, their leadership and decisions. Decision-making in Maasai society is more collective rather than individualistic and it is the village chairman that is expected to facilitate discussions related to village activities and decisions. A Maasai leader advising the research group advised that village chairmen would choose women who had experience participating in other community programs and were proactive in transferring information from programs to the community. It was expected that village chairmen would fairly choose appropriate women based on these criteria to represent their sub-villages. Feedback from the community indicated selection was fair in two villages while one village chairman was accused of selecting women based on personal interest (his wives and other relatives). It was not known if women ‘voluntarily’ accepted participation after being chosen by the village chairmen. Our experience in this Maasai community and particularly with the selection of community drug distributors for MDA for trachoma control in the same setting, people are willing and honoured to participate in programs intending to improve the lives of their community. There is a respect and prestige associated with attending programs. It should be noted that unintended coercion is possible when selection of participants is done by a community member. The decision to have the village chairmen central to the selection process was to respect the local hierarchical system and culture. Despite positive feedback on the intervention from all women who participated, the researchers were unable to assess if there was any coercion to participate.

The workshop was conducted at the Il Donyo village office in September 2017. Women were given transport allowance and all whom were invited attended the workshop. The workshop was conducted by two Maasai research assistants (JM, JL), supported by the lead researcher, who were familiar with the community and whom had established a rapport with leaders and community members during the course of the fieldwork pre-empting this engagement activity.

The use of photos taken in a photovoice project requires consent from third parties captured in the images [23]. Consent allows for use of the photos to raise public awareness and to share with collaborators. An additional goal of this photovoice intervention was to display the photos taken by the women at the weekly village market day to raise awareness of trachoma control among community members, local leaders and health officials. The issue of asking workshop participants to obtain consent was discussed with Maasai researchers and village leaders. It was strongly advised not to do so. They advised it would not be possible due to low literacy of the entire community. Additionally, it was thought to lead to potential mistrust in the lead researcher and the project.

The workshop was delivered in Maa (Maasai language) by two Maasai research assistants. The lead researcher designed the curriculum of the participatory workshop using the local understandings of the disease as a foundation gained from previous research (see [17]). The workshop was approximately 3 ½ hours and covered basic information on causes and pathology, transmission, signs and symptoms, treatment and prevention. Women were then asked to go back to their villages as advocates or ‘ trachoma control ambassadors’; providing education, advising and mobilizing the community. In this role as ambassadors, women shared their knowledge from the workshop with other women in their village and facilitated discussions on control measures within their socio-cultural context. The following participatory methods were used in the workshop.

### Giant fly models

Giant stuffed, *M. sorbens,* were used to show transmission of chlamydia trachomatis. Baby powder, representing chlamydia trachomatis, was put around the eyes of women. It was demonstrated that when the giant fly landed on their eye it got some baby powder on its feet and then flew off and landed on another woman’s eye and left some baby powder on her eye.

### Story telling

Issues around trachoma infection and treatment were conveyed through a story telling session, which used an adapted version of the story of Kokwana from the book “a Village Struggles for Eye Health” [24] translated into Maa.

### Video demonstration

A short video by Sightsavers, “Leaky Tin: A Simple Solution”, was shown and simultaneously translated in Maa to show a simple and effective way to wash faces and hands using minimal amounts of water. This demonstrates the use of a container with a hole poked into the bottom. When filled with water hand and face washing can be done with the water slowly trickling down. The hole can be plugged with a thorn when not in use. Since the video was of Maasai from Kenya it was thought to have more of an impact with members of their own community addressing trachoma prevention than seeing people outside their community on the video [25].

### Discussion groups

Women were asked to go back to their villages as trachoma control ambassadors by sharing knowledge from the workshop with other women in their village and facilitating discussions on control measures within their socio-cultural context. Through three small discussion groups based on the villages they came from, women planned ways of disseminating knowledge from the workshop to other women in their village. All three groups decided that the two women selected from each sub village would ‘co-teach’ the knowledge they gained from the workshop at a meeting of mothers and grandmothers in their sub-village.

### Photography

**Fig. 2:**
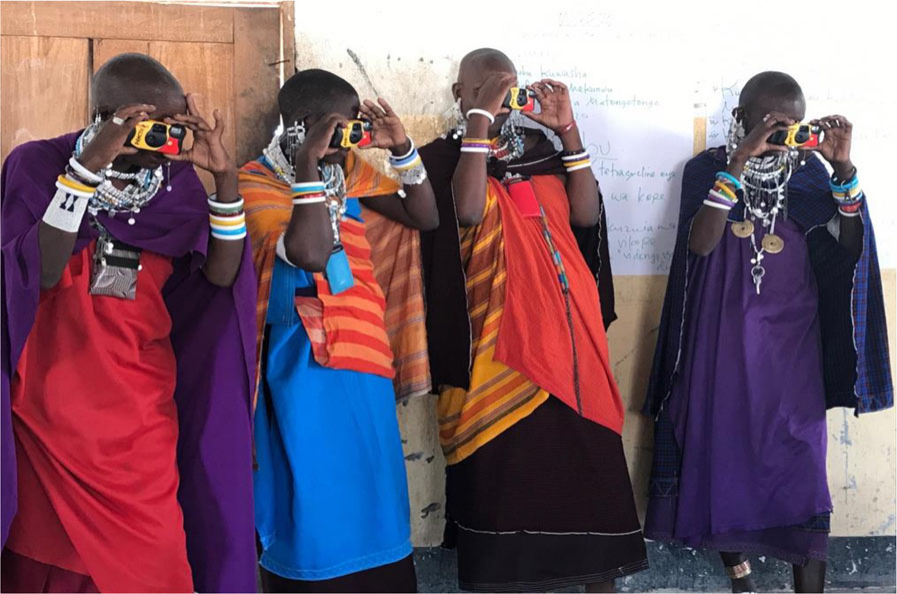
Participants practicing use of disposable cameras

Women were given disposable cameras to document their efforts as trachoma control ambassadors and the challenges raised around trachoma control. They were taught how to use the disposable cameras, what types of images they can capture and the ethics of photography including seeking permission prior to photographing people or private property and respecting people’s choices not to be photographed (Fig. 2). Women were asked to photograph people, activities and things that can convey their efforts as trachoma control ambassadors including successes and challenges. Women were encouraged to consider the confidentiality of people and places being photographed by taking metaphorical photos, aimed at using creative ways to depict a situation symbolically [26]. Examples given at the workshop included photos of soap and water rather than a person bathing; or clean clothes drying on a line rather than a woman washing clothes.

The day of and the following day of the workshop, the two Maasai research assistants, one village leader and the lead researcher visited the homes of each participant to meet their husband or the male elder of the *enkang*^1^ to seek permission for woman to use cameras to document their experience as trachoma control ambassadors. All agreed for them to use cameras. The decision to include husbands or heads of household was an afterthought when women at the workshop requested this be done to explain why a woman in their home has a camera and will be taking photos. The women wanted this additional permission to carry out the intervention.

A week after the workshop, the research assistants and lead researcher again visited each woman to follow up on their progress as trachoma control ambassadors and to make sure they were comfortable using the cameras. Women were asked to return the cameras to the research team two weeks later on market day. All 20 cameras were returned. The cameras were sent for film development.

Five weeks post-workshop, the lead researcher and research assistants held a follow up meeting with the women. Each woman received their set of photographs to keep. The women were asked to select two photos to discuss with the group- ideally one showing a success and one a challenge in their efforts as trachoma control ambassadors. The lead researcher, led a group discussion whereby each woman individually shared their photos with the group. Woman described their two photos and all women engaged in discussions related to the photos based on SHOWeD [27]: What do you **S**ee here?, What is really **H**appening here?, How does this relate to **O**ur lives?, **W**hy does this situation, concern, or strength **e**xist?, What can we **D**o about it?

## Intervention outcomes

The most commonly shared photos were of children either washing their faces or displaying clean faces with no flies. Women shared photos of individuals cleaning up trash or human and animal faeces, cleaning clothes and displays of ‘leaky tins’ at their *enkang*. Some women shared photos of themselves conducting meetings with other women in their village to educate them on trachoma. Very few women shared photos of challenges but of those that did the photos showed traditional practices of treating trachoma-like symptoms [17] and of animal carcases. The workshop only mentioned faeces as a source of flies and despite this, women concluded on their own that animal carcases were a source of flies. This is a very real source of flies in this community with a lot of carcases of wildlife and donkeys close to their *enkang*.

### Empowerment

Although empowerment was not systematically measured, it was observed that women articulated empowerment as participants in this public engagement intervention. Budig’s (2018) areas of empowering women is used to reflect on its application through participation in photovoice.

#### A gain in knowledge and skills

Women gained knowledge about trachoma from the workshop. Photovoice provided a means to share how women transferred knowledge from the workshop to their community and the response from the community This was evident from the follow up visit to their homes as well as the photos they shared particularly those taking action to reduce risk factors in their homes. There was evidence through the photos of women cleaning up animal faeces at their *enkang,* erecting leaky tins for hand and face washing and increased face washing for children and adults. Photovoice provided a personalized perspective that researchers or NGOs would not otherwise experience- images inside people’s homes, engaging in social activities, and documenting behaviours that words could not fully capture. Women also gained skills of using a camera.

#### Changes in self-perception

**Fig. 3:**
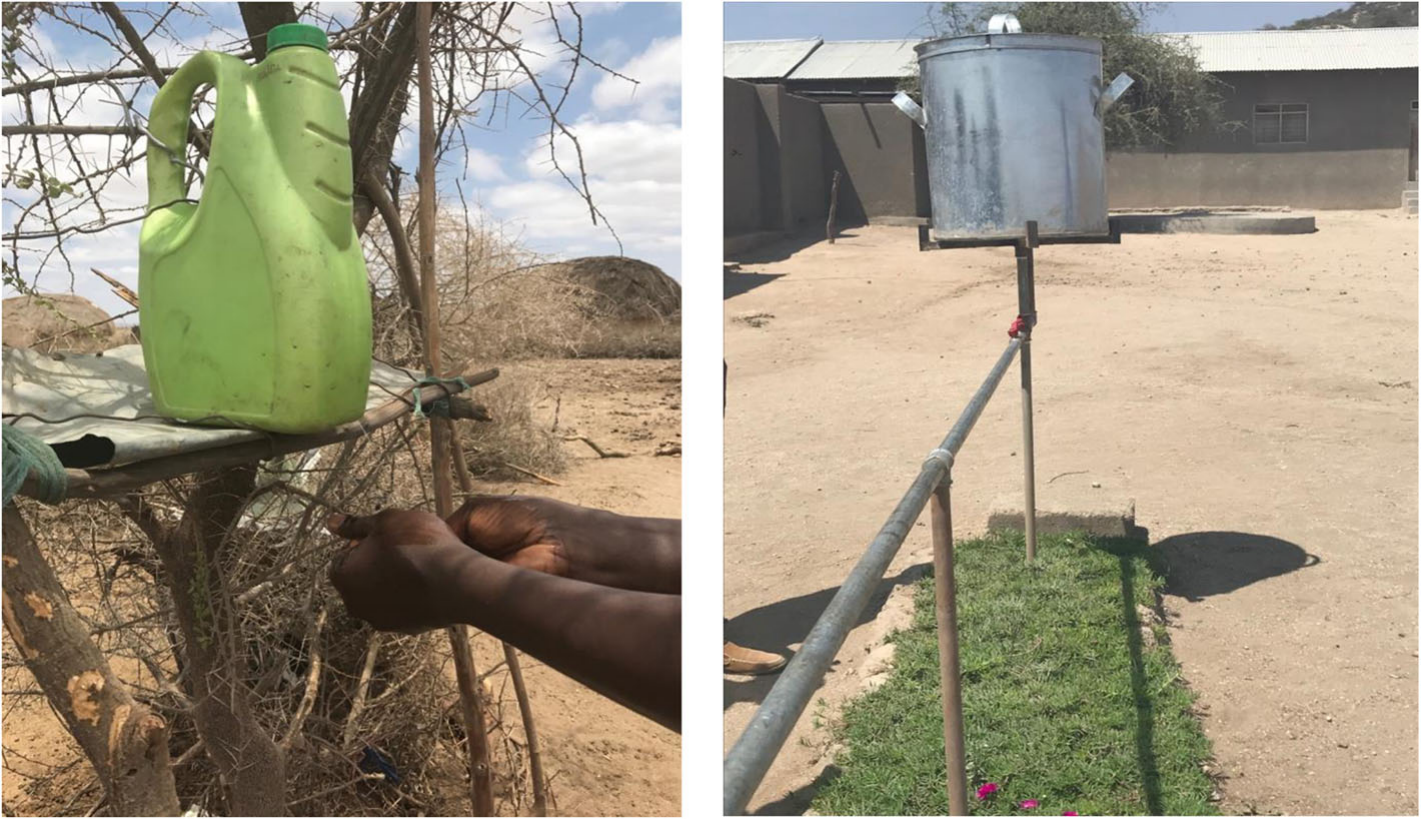
‘Leaky tins’ installed at [1] a participant’s boma, and [2] local primary school after the trachoma workshop

Women felt proud to be selected by male community leaders to take on the responsibility of representing their sub-village and acquiring new knowledge. They felt valued as experts within their community when they disseminated their newly acquired knowledge. Women were agents of change in their community by spreading the trachoma education they received to other women in their communities. Conversations and photographs demonstrated participants effectively stimulated social change. The most obvious example was the implementation of leaky tins in nearly all *enkang* visited by the research team one-week post-workshop. Women and men proudly guided the researchers to the newly erected hand and face washing station. Additionally, the local boarding primary school constructed a larger, more studier version made from metal pipes and of which numerous children can wash simultaneously (Fig. 3).

At the 5-week post workshop meeting, women showed excitement and pride when they received the photos they took and were pleasantly surprised and expressed appreciation when it was explained that the photos were theirs to keep.

#### Access to and use of resources

Women were given the opportunity to use cameras, a first time for all of the women. When first given the cameras, they were unsure which way to hold them or what the mechanism was to capture an image. Through demonstrations and practice they proudly mastered locating an image, focusing on it and pressing the shutter-release. The women were being trusted to provide information with cameras which added additional value for them. Through gaining knowledge to control trachoma, women were trusted by husbands to utilize household resources to aid in control measures such as containers for leaky tins, purchasing soap for washing bodies and clothes and use of limited water for preventive techniques.

### Photography and public trust

Documenting community reality and discussing community change are walking a fine line of politics [28]. Participants may face uncertainty or experience unintended consequences. Participants reported that community members questioned the real intentions of using cameras to capture the livelihoods of their people. They questioned if the *mzungu* (white person) researcher was going to sell the photos or use them for tourism. Photographic safaris are a source of income for Maasai where they often welcome tourists into their *enkang* for traditional dances and pose for photos on the side of roads. Despite the income generated, Maasai explained that photos taken by tourists is perceived as exploitation of their people. One elder discussed a recent incident in which tourists in Sinya, had photographed him while bathing in a dam. “Photography is an aggressive act, at least in the context of international tourism, where it is a means of dominating the object” [29, 30]. Participants were able to clarify the goals of the use of the cameras to many community members but some still did not understand nor trust the photographer’s intensions and use of the photos. This was evident in some photos, that depicted people walking away, hiding their faces and, in once case, of a man waving a stick at the photographer. All women said they faced challenges related to the cameras but most felt confident their clarifications were well received. “There’s always a few people that have doubts so its ok” [woman participant 6].

The ethics of photography was discussed in the workshop, including requesting permission to take photos and respecting people’s requests not to have their photo taken. It was noted at the workshop to take metaphorical photos; photos did not need to include people or show their faces in order to display their efforts as ambassadors or the successes and challenges around trachoma control. Despite the efforts to avoid problems in the community around the use of cameras, there were few unintended or negative consequences.

## Discussion

As a socially and politically marginalized population it was anticipated that putting Maasai women behind the camera would be empowering, provide an insider perspective of their livelihoods and an effective means to promote trachoma awareness within the community. This public engagement activity explored the use of photovoice as a tool to empower women to be agents of change for trachoma control in their communities. Health education accompanying health service delivery programs including trachoma control programs is inadequate and often ineffective due to budget and time constraints [20, 31]. Community level health behavior change requires moving beyond didactic methods to include skill development, knowledge acquisition and active community involvement. An informal participatory approach to health education is effective; “a process that enables, encourages, supports and facilitates, but does not impose” [32]. Participatory methods, including use of photovoice, used in this project were effective beyond knowledge transfer and awareness raising in that it promoted social and emotional connectedness through a shared experiences, a sense of solidarity.

The use of photovoice was an effective tool for motivating and facilitating community discussion about trachoma control and ultimately empowered women engaged in the intervention. Similarly, photovoice used in other Maasai contexts were effective in engaging Maasai in discussions prompted by their own photography on topics of women’s perceptions of development [33] and animal health needs [34]. Yet unlike prior photovoice projects in this community, this was the first time that photovoice was used for public engagement. Some of the advantages of using photovoice specifically with Maasai women includes documenting their socio-cultural settings including their risk behaviors; to allow the community to showcase the assets of their social network [35] and possibly stimulating social action by encouraging women to be proactive around their community’s well-being [28]. The researchers recognize the ethical tension of engaging this vulnerable population in this participatory method of generating knowledge. Yet with recognizing the ethics of photovoice and culturally appropriate engagement of community leaders prior to the photovoice project, any unintended consequences were greatly reduced .

In a patriarchal culture such as the Maasai and coupled with women being at the forefront of health issues in their community [36], women’s’ voices are imperative. Photography of the Maasai is embedded in their culture on different historical levels. The Maasai have been depicted as exotic, noble savages in various visual images from ‘trade cards’ in the late nineteenth century to today contemporary coffee table books. Considered a must see along with the ‘Big 5′ on photographic safaris in Tanzania and Kenya, Maasai are sought out to capture images of them adorned in beads and posing with spears [37, 38]. Yet, this intervention put the cameras in the Maasai’s hands giving them a sense of control and power in photography.

Feminist theory implies that power is held by those who voice, set language, make history and participate in decisions [39]. It honors local understandings which cannot be fully conceptualized from outsiders [40]. This intervention demonstrated that participation in photovoice in this context empowered Maasai women according to three areas described by Budig et al. [41]: [1] a gain in knowledge and skills, [2] change in self-perception and [3] access to and use of resources. Photovoice carried out by women intends to be by and with women rather than on women. It aims to honor women and values their experience and position in society [27]. Empowerment can include a change in how participants access and use resources or information; and the formation and potential of social relations [41, 42]. Empowerment has been described as a significant outcome in photovoice projects [43–45]. Although there are a number of factors that may contribute toward empowerment in these projects it is the totality of the method that includes a sense of trust in participants being photographers, a catalyst to explore and share lived experiences and feeling valued for their contribution toward social change.

Despite efforts to emphasize metaphorical photos, most photos depicted community members. Women discussed the enjoyment in taking photos of family and friends, something they had never done and the thrill of receiving the print photos of people they took themselves. Therefore, the researchers were unable to display the photos in public since consent from those being photographed was not given. As the photos were only in print format, they were given to the women who took ownership of them and the negatives destroyed by the lead researcher. In many cases women gave photos to the people captured in the image. Despite this inability to disseminate photos more broadly to the community, the participants were comfortable to discuss their experiences with the lead researcher and bring information back to the community. An additional benefit was the positive experiences in being given autonomy to photograph as they chose. Maasai reported similar experiences in other photovoice projects [33, 34].

### Lessons learned of using photovoice in resource poor-contexts

Informed consent was not obtained for this public engagement activity as it was viewed as a barrier to the community feeling comfortable and open to share and participant in activities; it was advised that it would affect trust in the intervention and the researcher. Informed consent in research is based on a western context focusing on individual rights, a sense of the ‘self’ and a legal protection [46, 47]. In Maasai communities, similar to many African contexts, emphasis is on community and collective decision-making [48, 49]. To respect this interdependence within the community, the research team obtained permission from community leaders to conduct the public engagement activity and from heads of households to allow women to take photos in the community. The disadvantage of not obtaining consent is that it does not allow the researcher to formally analyze and interpret the ‘data’ (in this case photos and transcripts of feedback session) nor display or publish the photos. Yet with reciprocity, being the aim of this public engagement activity and not research, the researchers were agreeable to not obtaining informed consent. It should be emphasized that it is the story and experience around the images that is more important than the images itself.

The lead researcher’s acceptance of the communities advice not to obtain third-party photo release forms and her ability to be reflexive about her own power in the project was critical to acknowledge the ethical context and safety of the participants [50]. There was flexibility from traditional ethics to adhering to situational ethics, seemingly more relevant in this community. Traditionally, decision making to participate and the concept of autonomy is based on self-determination. Relational ethics is based on the principles of “engaged interaction, mutual respect, embodied knowledge, uncertainty and vulnerability and interdependent environment” [51]. It views decision-making as being ‘with’ a participant, rather than ‘for’ a participant. From a relational perspective, the social, economic and political context of participants influences their decisions. Decisions are framed by their relations both within their community and with researchers; embedded in systems of power [52]. Interpreting autonomy in terms of individual choices without considering the context, potentially ignores a community’s socio-cultural system and adds additional power imbalances to participation. Relational ethics in photovoice has been used with vulnerable women [26] and is relevant to consider in the African context [48].

In photovoice the images including film negatives are considered the property of the photographer [23]. A researcher must obtain informed consent from the participant, “photographer”, as well as consent from third parties whose images are captured. In this intervention, printed images were handed over to the participants to keep and negatives of the prints destroyed by the researchers. This was discussed with community leaders to allow for transparency in the purpose of the activity.

Issues of respect for privacy are embedded in the ethics of capturing images. The act of capturing an image may invade one’s personal space in addition to invading privacy by capturing a personal moment. A photographer’s depiction of people or a situation may be portrayed differently in their photos, the use of photos and the description of photos from how a person or group of people see themselves and/or want to be seen. Although some argue that a photograph is no different from a fully written description [53] “such an action is not ethically neutral” [23]. The power of a photographer to produce, interpret and potentially benefit from images creates a vulnerability of those being photographed. With Maasai so often photographed in tourism, this activity shifted power to Maasai photographers and women discussed the pleasures in being on the other side of the camera. It was noted that giving this power to women in a patriarchal community required permission which were obtained from the male heads of households for each participant. Women were taught the ethics of photography and to assure the safety of themselves and those in the community by taking metaphorical photos. This strategy was highly effective in minimizing the number of identifiable photos and minimizing pushback from people not wanting their photos taken.

Photovoice projects have used video cameras, digital cameras, disposable cameras and smart phones. Each has its strengths and weakness [54, 55]. A final reflection was the challenge of using disposable cameras in this photovoice project due to budget constraints, low use of smart phones and illiteracy in this community. Whilst the women had never used a camera before yet they found the simple cameras easy to use. However, it was challenging to develop the film as such services were not available in Tanzania. Instead the cameras had to be sent to UK for film development. This led to a three-week delay between collecting the cameras and holding a meeting with the women to disucss their photos and experiences. There was the possibility of lost to follow up of women or recall bias. Fortunately neither was an issue in this intervention as all women returned for the meeting and a combination of the photos with dialog between the women assisted in recalling their experiences.

## Conclusion

While this was not the first photovoice project in Maasai community [33, 34] it is the first focusing on health and specifically trachoma control. Although this project was not designed to collect research data to inform future interventions or policy, it was effective in empowering women to be change makers, to encourage them to have a voice in educating their communities on trachoma control. The project also provided a guided and systematic approach for the women to discuss openly what risk behaviours are reasonable to change and those that are challenging in their socio-cultural context. This public engagement intervention showcased an effective method to share with the public aspects of the ethnographic research conducted in their community. Through a mutual benefit, the researchers confirmed the use of photovoice as a positive tool to engage the public and the community demonstrated engagement in the research topic of trachoma control and empowerment among women that participate. Effective health promotion occurs within the community. Photovoice enabled women to promote positive health behaviours and to initiate conversations around trachoma. Photovoice should be considered for future public engagement as a communication tool on health issues and to empower women to be ambassadors for health promotion.

## Data Availability

Data sharing not applicable to this article as no datasets were generated or analysed during the current study.

## Abbreviations

MDA: Mass Drug Admnistration
NTD: Neglected Tropical Disease
SAFE: Surgery for trichiasis, Antibiotics, Facial cleanliness, Environmental improvement
WHO: World Health Organization

## Footnotes

1 An *enkang* is a homestead or joint residential unit composed of a number of households. They are headed by one male and in a polygamous culture, each wife with her children have a hut within the boma.
